# Editorial: The role of multi-omics variants in tumor immunity and immunotherapy

**DOI:** 10.3389/fimmu.2022.1098825

**Published:** 2022-11-29

**Authors:** Shuang Chen, Hui Xu, Chunguang Guo, Zaoqu Liu, Xinwei Han

**Affiliations:** ^1^ Department of Interventional Radiology, The First Affiliated Hospital of Zhengzhou University, Zhengzhou, Henan, China; ^2^ Center of Reproductive Medicine, The First Affiliated Hospital of Zhengzhou University, Zhengzhou, Henan, China; ^3^ Medical School of Zhengzhou University, Zhengzhou, Henan, China; ^4^ Interventional Institute of Zhengzhou University, Zhengzhou, Henan, China; ^5^ Interventional Treatment and Clinical Research Center of Henan Province, Zhengzhou, Henan, China; ^6^ Department of Endovascular Surgery, The First Affiliated Hospital of Zhengzhou University, Zhengzhou, Henan, China

**Keywords:** multi-omics variants, tumor immunity, immunotherapy, precision medicine, cancer

The immune system exerts a crucial influence on the antitumor response *via* the complicated interaction of different immune cells and molecules. Cancer immunotherapy has reversed the traditional oncology treatment landscape with high expectations in tumor management, yet only a small subset of patients who enter care with immune “hot” tumors can truly derive clinical benefits from immunotherapy (1, 2). Given the complexity of immune processes and the heterogeneity of patients’ immune responses, it is imperative to optimize the selection of patients appropriate for immunotherapy and precisely implement tailored therapeutic regimens in the clinical setting (3). The dawn of large-scale sequencing and diversified high-throughput biomolecular technologies makes it possible to quantify the highly dynamic and interactive multi-molecular layer systems, which are involved in cancer genesis and evolution. The complementary effects and synergies of cross-talks among divergent omics layers (including genomics, transcriptomics, proteomics, epigenomics, metabolomics, etc.) can only be captured by the integrated analysis from multiple molecular layers (4) (**Figure 1**). Thus, the traditional strategy of characterizing specific biological processes from the single omics level may miss crucial information. The combination of multi-omics variants in immunological studies allows for a holistic insight into molecular layer interplay and comprehensive impacts on the disease course, which paves way for enhancing immunotherapeutic efficacy (5). Deep excavation and wide application of massive omics data in tumor immunology have spurred new advances in the precision medicine paradigm. The integrated analysis in multi-omics studies facilitates the full elucidation of molecular underpinnings within tumor immunity, the systematic exploration of subtypes, the efficient identification of specific biomarkers, the accurate prediction of clinical evolution, and the customization of individualized treatment strategies (6–9).

**Figure 1:**
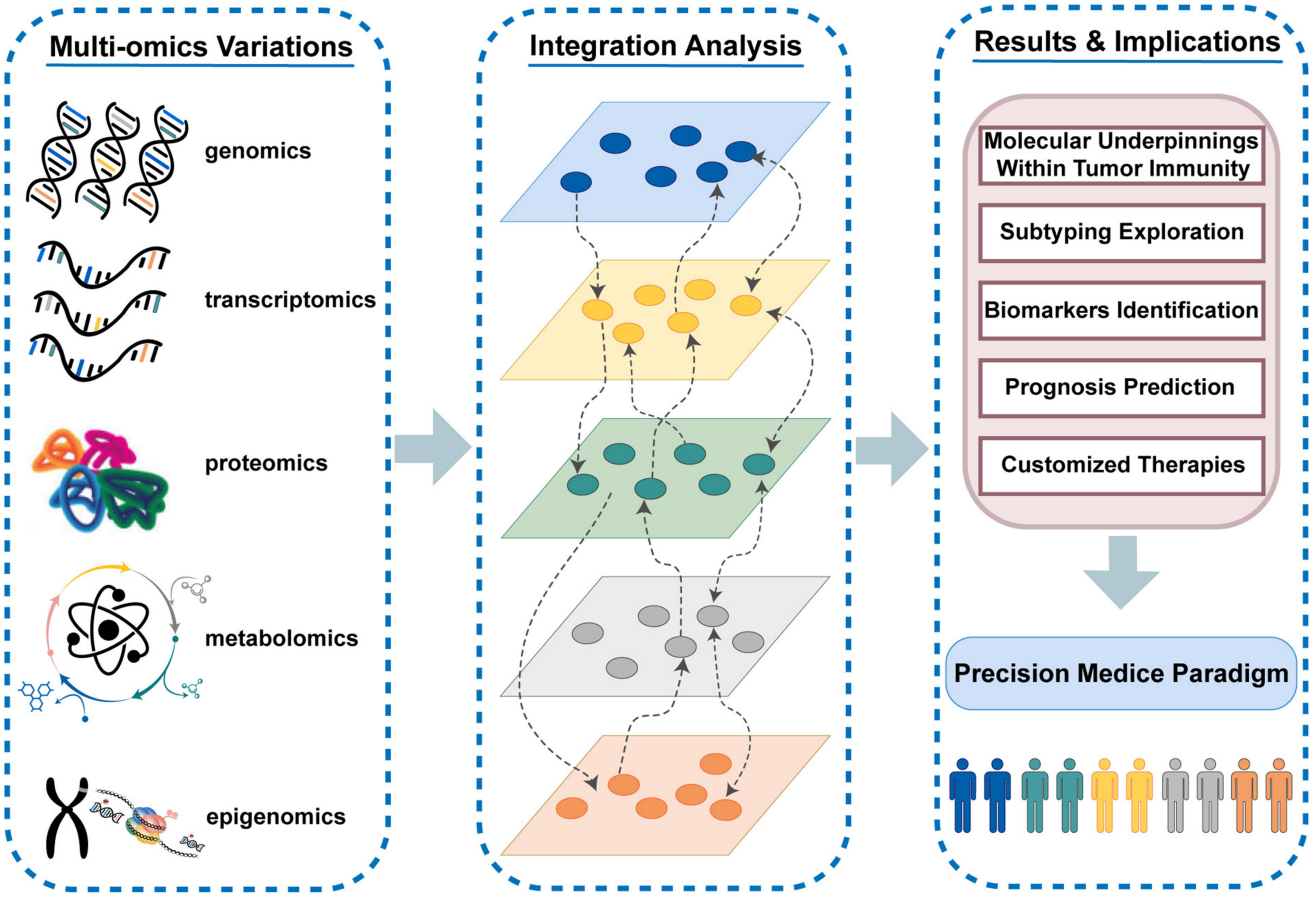
The highly dynamic and interactive multi-molecular layer systems and the vital role of multi-omics variants in tumor immunity and immunotherapy.

A total of 8 articles from 64 authors focusing on the combination of multi-omics analysis and immunotherapy were accepted in this Research Topic, addressing the integral role of multi-omics variants in tumor immunity and immunotherapy. In this collection, 3 manuscripts highlighted the implications of deciphering heterogeneity across distinct subtypes for specific cancer from a multi-omics perspective, leading to novel guidance for patient-centered cancer management. In light of the potential association of copper metabolism and cuproptosis-related genes (CMCRGs) with immune dysfunction, Liu et al. established two well-characterized molecular subtypes for hepatocellular carcinoma (HCC) based on the prognosis-related CMCRGs. Two heterogeneous subtypes could distinguish the clinical prognosis, immune landscape, and genomic variation of HCC. Furthermore, the high copper-related subtype displayed better sensitivity to targeted drugs and poor susceptibility to immunotherapy, thus providing a reference for personalized therapies. According to necroptosis-associated long non-coding RNAs (lncRNA), Hu et al. decoded two subtypes of HCC with profound heterogeneity in prognosis, biological function, clinical characteristics, genomics, tumor immune microenvironment (TIME), and drug sensitivity. Integrative analysis of diverse omics facets revealed the significance of lncRNA and necroptosis in antitumor effects and threw light on precision care for stratified HCC patients. Necroptosis-related genes were recruited by Zhao et al. and three novel necroptosis phenotypes for lung adenocarcinoma (LUAD) were subsequently developed. Discrepancies among three necroptosis patterns in clinical features, immune infiltration, and somatic mutational characteristics were evaluated, which unveiled the pivotal role of necroptosis in shaping TIME. The construction of tumor prognosis-related biomarkers was the subject of 4 articles. Founded on mast cell-related genes, Chen et al. proposed a prognostic model for gliomas and screened *DRG2* as a characteristic gene for therapeutic response and survival outcome prediction. Besides, mast cell-associated immune escape mechanisms in glioma were also probed from multi-omics dimensions. Immune-related genes for Wilms tumor were exploited by Tian et al. to establish an immune-related prognostic signature, whose properties were further excavated from a multi-omics perspective. This immune signature was demonstrated to be a predictive biomarker of clinical immune response with desirable efficiency. Mutation profiles of 38 genes along the RTK/RAS pathway in LUAD, as well as their close relationships with immunity and prognosis, were systematically evaluated by Yin et al. A nomogram survival model was built from multidimensional research, and several latent prognostic predictors were identified. This combined mutation-immune strategy would serve as a theoretical basis for the combination of targeted therapy and immunotherapy. Grounded on TIME and genomic instability (GI) related factors, Xu et al. constructed a four-factor prognostic risk model for cervical cancer and screened *RIPOR2* as a prospective GI-related immune biomarker for forecasting a better prognosis. Lastly, 1 paper spotlighted the application of multi-omics in the single-gene study across pan-cancer. Considering the great cancer therapeutic potential of copper-induced cellular death, termed cuproptosis, Xu et al. delved into cuproptosis core gene *FDX1* in pan-cancer *via* multi-level exploration including transcriptomics, proteomics, epigenomics, genomics, etc. Linked with prognosis in various cancers, *FDX1* was proven to be a promising immunotherapeutic predictor and novel therapeutic target.

## Conclusion

In aggregate, 8 manuscripts on this Research Topic cover a multitude of hot topics in cancer immunotherapy, illuminating the indispensable role of multi-omics variants in tumor immunity and immunotherapy. These studies integrate a great deal of information from multi-omics layers to deepen comprehension of cancer subtype heterogeneity, identify prognostic biomarkers for specific cancer species, as well as dig deeper into the value of single genes in pan-cancer research, which will ultimately throw insights into precision medicine modalities.

## Author contributions

All authors listed have made a substantial, direct, and intellectual contribution to the work and approved it for publication.

## Conflict of interest

The authors declare that the research was conducted in the absence of any commercial or financial relationships that could be construed as a potential conflict of interest.

## Publisher’s note

All claims expressed in this article are solely those of the authors and do not necessarily represent those of their affiliated organizations, or those of the publisher, the editors and the reviewers. Any product that may be evaluated in this article, or claim that may be made by its manufacturer, is not guaranteed or endorsed by the publisher.
